# Underwater and Surface Swimming Parameters Reflect Performance Level in Elite Swimmers

**DOI:** 10.3389/fphys.2021.712652

**Published:** 2021-09-01

**Authors:** Robin Pla, Gauthier Poszalczyk, Cyrine Souaissia, Fabrice Joulia, Alexandre Guimard

**Affiliations:** ^1^French Swimming Federation, Clichy, France; ^2^Institut de Recherche bioMédicale et d'Epidémiologie du Sport, IRMES, Paris, France; ^3^Université Sorbonne Paris Nord, Hypoxie et Poumon, H&P, INSERM, UMR 1272, Bobigny, France; ^4^Département STAPS, Université Sorbonne Paris Nord, Bobigny, France; ^5^National Health Service (NHS) Grampian, Aberdeen Royal Infirmary, Aberdeen, United Kingdom; ^6^Center for Cardiovascular and Nutrition Research CV2N, Aix Marseille Université, INSERM 1263, INRAE 1260, Marseille, France

**Keywords:** apnea training, breath-holding, immersion, freestyle swimming, elite swimmers, dolphin kick, apnea plan

## Abstract

Although the role of underwater phases is well-known, no study has taken an interest in describing and quantifying the distance and time spent in apnea as a condition for optimal performance. This study aimed to investigate the impact of time and distance spent underwater and surface parameters on the swimming performance of elite swimmers. The performances of 79 swimmers in 100-m freestyle were analyzed (short-course). The underwater and spatiotemporal parameters of three groups have been recorded: finalists of the 2018 World Swimming Championships (WORLD), French swimmers who reached a 100 m performance time under 50 s at the 2018 French National Championships (UND50), and those who reached a 100 m performance time above 50 s (UP50). The WORLD group spent more distance underwater (37.50 ± 4.92 m) in comparison with UND50 (31.90 ± 4.88 m, *p* < 0.05) and UP50 (31.94 ± 4.93 m, *p* < 0.01) groups. The total percentage of non-swimming time was higher for WORLD (39.11 ± 4.73%) vs. UND50 (34.21 ± 4.55%, *p* < 0.05) and UP50 (33.94 ± 5.00%, *p* < 0.01). In addition, underwater speed was higher for WORLD (2.54 ± 0.05 m/s) compared with UND50 (2.46 ± 0.09 m/s, *p* < 0.05) and UP50 (2.38 ± 0.11 m/s, *p* < 0.01). Three parameters among the underwater phases (i.e. distance underwater, speed underwater, and total percentage of non-swimming time) determine the 100-m freestyle short course performance. These data suggest an appropriate focus on specific apnea training to improve underwater skills during short-course swimming performances.

## Introduction

Swimming competition analysis is highly documented, and numerous studies have investigated the features of spatiotemporal parameters during swimming competitions to determine their influence on the performance of swimmers (Craig et al., 1985; Huot-Marchand et al., 2005; Hellard et al., 2008).

A swimming race includes the swimming phases and the so-called non-swimming phases, namely, the start and the turns, such as underwater swimming segments. While, the work of Tor et al. remains the link between start time and swimming performance for high-level swimmers (Tor et al., 2015), a recent paper has highlighted the correlation between the performance and the start and the turns, especially for the 100 m races (Morais et al., 2019a). Furthermore, the time after 15 m after each length depends on under or above water swimming (Arellano et al., 1994; Veiga et al., 2014a, 2016). During 200 m races, the 15 m time after the start is better for the swimmers to cover in apnea large underwater distances (Veiga and Roig, 2015), benefiting from the reduction of wave drag under the water (Vennell et al., 2006).

According to the literature, the duration of underwater swimming seems to be more related to the performance in 200 m than in 100 m events (Veiga and Roig, 2015). The modifications on the start or turn distances (especially in the last turn) could represent the overall time improvements of a practical importance for 200 m elite swimmers (Veiga et al., 2016). Conversely, in these 100 m events, the average velocity of these underwater sections seems to be a key for the race performances. Indeed, despite not spending longer underwater distance, the faster swimmers at the World Championships 100 m events traveled with faster velocities during the freestyle and breaststroke start than slower swimmers (Veiga et al., 2016). Changes in the start or turn velocities could represent moderate time improvements in most of the 100 m events (Veiga et al., 2016). In short-course events, the greater contribution of non-swimming phases could emphasize a more significant effect on the performance where underwater phases can represent up to 60% of the race distance (FINA rules).

The underwater phases require high skills when applying the dolphin kick technique, since the leg extension from above the ankle to the toes plays a huge part in producing a propulsive force (von Loebbecke et al., 2009). It depends on the importance of the trunk undulation (especially the chest bending) (Nakashima, 2009), and the ankle muscle strength and flexibility (Willems et al., 2014; Shimojo et al., 2019).

In addition, swimming such a distance underwater enhances the interest toward the physiological repercussions on the swimmer, during the non-swimming phases in an apnea situation. Apnea induces a typical cardiovascular response called a diving response, such as bradycardia (Foster and Sheel, 2005), which can compete with exercise tachycardia (Wein et al., 2007; Alboni et al., 2011) during the underwater apnea stages. If some swimming studies have focused on the physiological repercussions of apnea (vs. breathing) in a situation of surface swimming (Guimard et al., 2014, 2017, 2018), to our knowledge, one study has considered the underwater physiological aspects but without determining the influence of the non-swimming phases (Rozi et al., 2018).

The non-swimming phases seem essential during a 100 m short event, but these phases are most often used and worked on empirically by the swimmers and coaches. This study aimed to analyze the underwater and surface swimming parameters according to the level of performance of elite swimmers in a 100 m freestyle competition, and thus to highlight the strategies used in a competitive situation.

## Materials and Methods

### Participants

Data from the 100 m freestyle have been recorded in male swimmers for two events: (1) the 2018 Short Course World Championships (WORLD) (*n* = 8) in Hangzhou (China) and (2) the 2018 French National Championships (*n* = 71) in Montpellier (France). The two groups were divided among the French National Championships: the swimmers who reached a 100 m performance time under 50 s (UND50) (*n* = 21) and those who reached a time upper to 50 s (UP50) (*n* = 50). The swimming speed value of 2 m/s seems a threshold value to reach. The procedures have been conducted with adequate understanding and written consent of the participants and the study was carried out by the Code of Ethics of the World Medical Association (Declaration of Helsinki).

### Video Collection

Three cameras (Sony FDR AX700, Tokyo, Japan) have been positioned perpendicularly to the length axis of the pool, at 5, 12.5, and 20 m from the starting block. The film of each race has been analyzed with dedicated software to calculate the performance metrics of each swimmer.

### Data Collection and Data Treatment

Race analysis software (Espadon, Actriss, Brest, France) was used for the calibration and the image was processed by manual digitalization, to obtain the time and distance of each stroke cycle as previously described (Hellard et al., 2008). The software converts the pixels into distance (meters, SI units), based on the calibration made using four poolside marks in the swimming pool (wall and lane). The video analyst made this calibration and then manually digitalized the head position at the beginning of each stroke cycle (right-hand entry) to obtain the time and distance of each stroke cycle, as already described by Hellard et al. (2008). The speed, stroke rate (SR), stroke length (SL), and stroke count (SC) were computed for each 50 m lap. In order to measure all the stroke variables, the time and spot of the first and last arm water entry for each lap was calculated, giving the beginning and the end of each “swim-time” period. SC is the total number of arm entries on the water surface. SL is calculated by dividing the free-swimming distance by SC. SR is obtained by dividing the free-swimming time by SC. SI (stroke index) is the product of the swimming speed (lap distance divided by lap time) and SL. The underwater distance is equal to the distance between the wall to the head of the swimmer at the moment of stroking resumption (Veiga and Roig, 2015). The underwater time (expressed in seconds) is equal to the time to cover that underwater distance. The underwater speed is equal to the official split time at the wall contact and the split time at the stroking resumption (Veiga and Roig, 2015). Finally, the non-swimming time (expressed in percentage) is equal to the total time minus the free-swimming time, divided by the total time. In other words, the non-swimming time includes all the non-swimming segments of the race as start, underwater, and turns.

The assessment of performance metrics—used with the help of the software—was managed by a video analyst with a scientific background who is part of the French swimming staff.

### Statistics

For all the variables, descriptive statistics (mean and standard deviation) were performed. Normal Gaussian distribution of the data was verified by Shapiro–Wilk's test and homogeneity of variance by a modified Levene's test. The differences between the groups (UP50 vs. UND50, UP50 vs. WORLD, and UND50 vs. WORLD) were compared using an unpaired Student's *t-*test. Null hypothesis was rejected at *p* < 0.05. The statistical analyses were undertaken using the software package STATISTICA (version 8.0, Statsoft, Tulsa, OK, USA).

## Results

The mean swimming performance was higher for the swimmers of WORLD with a total time matching 97.48% of the world-record, whereas the swimmers of UND50 and UP50, respectively, represented 91.27% and 88.27% (*p* < 0.001). All the swimmers of WORLD reached a 100 m performance time under 47 s.

**Figure 1:**
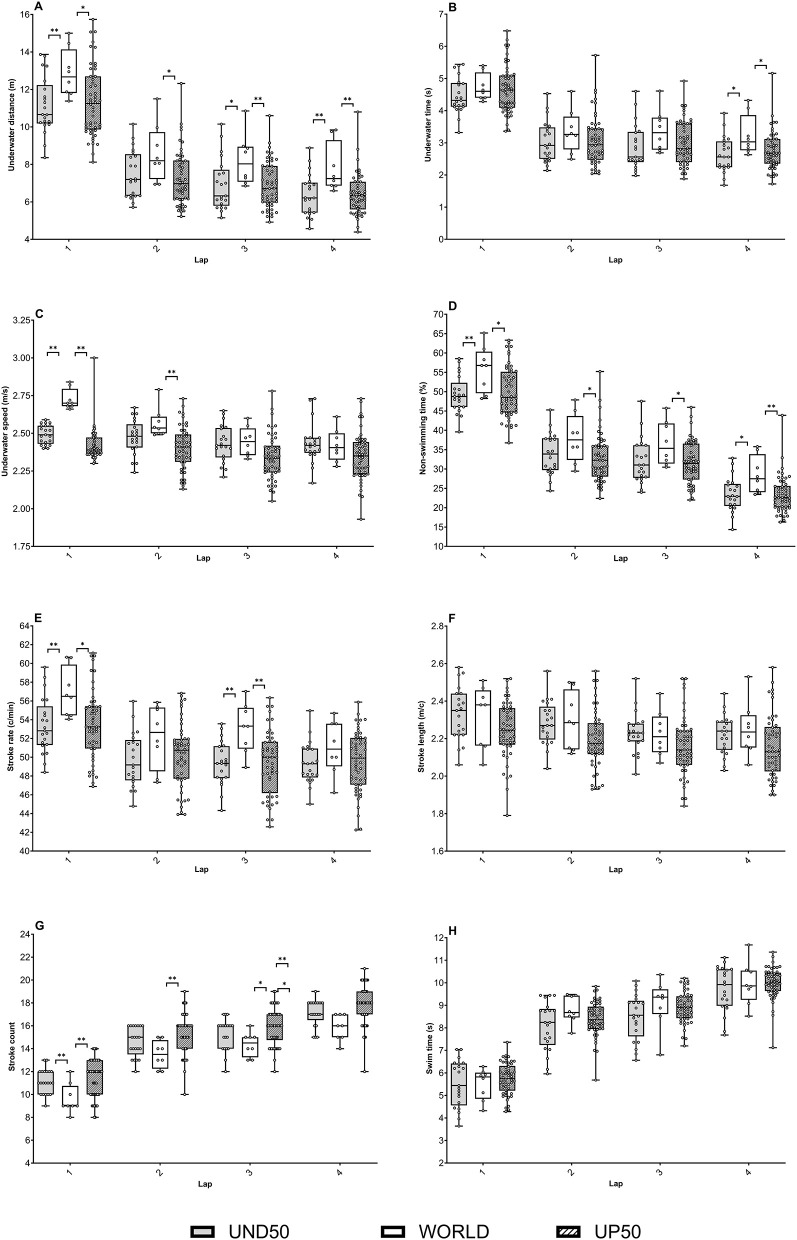
Underwater and spatiotemporal parameters for the world finalists (WORLD), French swimmers under 50 s (UND50), and French swimmers upper to 50 s (UP50) over each lap of the 100-m freestyle race. **(A)** Underwater distance, **(B)** underwater time, **(C)** underwater speed, **(D)** non-swimming time, **(E)** stroke rate, **(F)** stroke length, **(G)** stroke count, and **(H)** swim time. Significant difference between the WORLD vs. UND50 and the WORLD vs. UP50: *p < 0.05, **p < 0.01.

Table 1 provides a comparison of the underwater and surface strategies for the 100-m freestyle swimming among the swimmers of WORLD, UND50, and UP50. Figure 1 details the differences among the groups for each lap of the 100 m freestyle race.

**Table 1:** Comparison of mean ± *SD* for performance, underwater and spatio-temporal parameters among the world finalists (WORLD) vs. French swimmers under 50 s (UND50) vs. French swimmers upper to 50 s (UP50) during the 100 m freestyle.

		**Performance**		**Underwater parameters**			**Spatio-temporal parameters**		
**Group**		**Total time (s)**	**Total underwater distance (m)**	**Mean underwater time (s)**	**Mean underwater speed (m/s)**	**Non-swimming time (%)**	**Mean stroke rate (c/min)**	**Mean stroke length (m/c)**	**Total stroke count**
WORLD	Mean	46.10	37.50	14.71	2.54	39.11	53.32	2.27	53.38
	SD	0.40	4.92	2.10	0.05	4.73	2.54	0.13	3.62
UND50	Mean	49.24***	31.90*	13.02	2.46*	34.21*	50.46**	2.27	58.14**
	SD	0.59	4.88	2.29	0.09	4.55	2.39	0.11	4.23
UP50	Mean	50.91***^###^	31.94**	13.50	2.38**^##^	33.94**	50.70*	2.19^#^	60.52**
	SD	0.73	4.93	2.45	0.11	5.00	3.03	0.14	5.91

Significant difference between WORLD vs. UND50 and WORLD vs. UP50: ^*^p < 0.05, ^**^p < 0.01, and ^***^p < 0.001. Significant difference between UND50 vs. UP50: ^#^p < 0.05, ^##^p < 0.01, and ^###^p < 0.001.

The total underwater distance (m) was higher in the WORLD compared with UND50 (*p* < 0.05) and UP50 (*p* < 0.01). Similar results were observed for the total non-swimming time (%), which was higher in the WORLD compared with the UND50 (*p* < 0.05) and UP50 (*p* < 0.01). The mean underwater speed was higher in the WORLD compared with the UND50 (*p* < 0.05) and UP50 (*p* < 0.05), while the UND50 had a greater mean underwater speed than UP50 (*p* < 0.01).

Figure 1 shows that the WORLD had a higher underwater distance than the UP50 for each lap of the 100 m, and it was more significant for the last two laps of the 100 m race (*p* < 0.01). Furthermore, WORLD had a higher underwater distance than the UND50 group in the first lap (*p* < 0.01), the third lap (*p* < 0.05), and the final lap (*p* < 0.01), whereas no difference was observed during the second lap.

The underwater speed was higher for the WORLD than for the other groups in the first lap (corresponding to the underwater phase after the dive start) (*p* < 0.01). However, no difference in underwater speed was observed between the UND50 and WORLD in the following three laps. The non-swimming time (%) was superior for the WORLD than for the UP50 in each lap of the race, whereas only the first and the fourth laps were higher for the WORLD than for the UND50.

Besides, the main outcome observed was the higher SR for the WORLD than for the UND50 (*p* < 0.01) and the UP50 (*p* < 0.05). In addition, the total SC was lower for the WORLD than for the UND50 (*p* < 0.01) and UP50 (*p* < 0.01). Also, Figure 1 highlights that there were no SL differences between the groups for each lap of the 100 m race. Finally, the WORLD had a higher SR than the UND50 and UP50 in the first lap (*p* < 0.01 and *p* < 0.05, respectively) and the third lap (*p* < 0.01).

## Discussion

The main outcome of this study investigation is that the difference in performance over 100 m is mainly in the non-swimming phases.

To our knowledge, this study was the first to examine the underwater and spatiotemporal parameters strategies depending on the three levels of swimming performance. Few differences occurred in the spatiotemporal parameters. The current study results show that the elite swimmers maintain a higher SR than the national level swimmers during the whole 100 m contest and both the groups obtain similar SL, while the UP50 has a smaller SL compared with the WORLD and UND50. This represents a new analysis of the swimming performance, since most of the studies have reported that the best swimmers have a greater SL than the other groups (Huot-Marchand et al., 2005). Such observation may challenge the idea of maximizing propulsion to improve swimming performance. Another study has already reported similar results to our work (Hellard et al., 2008), affirming that the best 200-m swimmers were much more distinguished by a higher SR, than a greater distance per cycle.

In our opinion, an SL “plateau” for the elite swimmers could exist, therefore to increase their swimming speed, swimmers would have to increase their SR. Otherwise, it is possible that the WORLD swimmers are able to sustain a higher SR because of their smaller SC, given by their larger underwater distance. As proposed by Alberty et al. (2008), the high metabolic demand required for an intense swim task is a restriction that could alter the stroke rate to maintain the required pace during the race. Thereby, the peripheral fatigue could be reduced during non-swimming phases by keeping the arms passive, to allow swimmers to keep up arm strength during the rest of the race. Ohkuwa and Itoh (1992) showed that the lactate in the blood predominantly originates from the arm muscle groups. Therefore, keeping a hydrodynamic position with a lengthened use of the leg muscles by the dolphin kicks technique could be a solution to have a positive impact on peripheral fatigue.

The results of this study highlight significant differences in the SC, particularly, during the first and fourth 25 m (as shown in Figure 1), not only with a smaller SC for the WORLD but also with a decrease in the total SC level. The differences noticed are linked to the larger underwater distance covered by the WORLD, allowing them to reduce the swimming distance. However, it should be noted that there were no significant results for the second and third turns. Besides, it is established that the last turn allows the speed of the swimmer to be maintained among the elite swimmers (Veiga and Roig, 2015). Mauger et al. (2012) have also shown that “fast-start” and “parabolic” (fast-start, speed decrease during the race, and a higher finish velocity) strategies are favored among the competitors in a 400-m swimming race. This statement confirms our results, with the same pattern as for the race management. Therefore, the two central laps do not seem to impact the number of arms strokes. The skills developed at the start of the race and during the fourth turn are probably the most advantageous for elite swimmers. Hence, it is important to suggest possible physiological adaptations to apnea in these swimmers as observed in apnea-trained athletes, who are able to perform dynamic apneas with attenuate fatigue signs (Joulia et al., 2009). Then, elite swimmers maintain a high-intensity level during the last immersion at the turn in order to delay the effects of fatigue.

Major differences between the groups are observed on non-swimming phases during the 100 m race. The WORLD spent more time in apnea covering a greater distance underwater and also spent more total distance underwater than the UND50 and UP50. It agrees with previous studies, and it can explain why international swimmers are faster than the others (Veiga et al., 2014a; Veiga and Roig, 2015). Thus, a high level of underwater skills would allow the swimmer to cover a long underwater distance to benefit from a reduction of wave resistance below the surface and thus increase the speed (Vennell et al., 2006). In addition, the time after 15 m (highly influenced by the underwater phase) depends on the level of competence (Arellano et al., 1994; Veiga et al., 2014a, 2016). It appears predominant in short-course swimming, where the underwater parts could represent up to 60% of the distance covered by the best swimmers in the world (FINA rules). However, it should not be generalized, because a lower level of competence supports a reduction in the time spent underwater after the start (Nazeer et al., 2016). We could explain such results by a reduced general control of the non-swimming phases and, therefore, a voluntary choice to swim faster and waste less time underwater. Additionally, the underwater distance differences between the groups clearly increase in the second part of the race. This is a clear characteristic of the higher skilled performers who maintain greater stability on underwater parameters as fatigue appears (Veiga et al., 2014b; Morais et al., 2019b). It could partly explain the high-level competency of the elite swimmers, who are able to keep control of the non-swimming phases, which widen the gap with national level swimmers, as the race progresses (Huot-Marchand et al., 2005).

Moreover, although the underwater distance is greater for the WORLD, the underwater speed is only higher after the dive (not the case for the next three turns of the race). This is probably due to the better start skills of the elite swimmers (Tor et al., 2015). In addition, we showed that, despite the same underwater speed, international swimmers than national swimmers moved a greater distance, suggesting that they are more efficient during underwater parts. This efficiency would depend on both the biomechanics and physiological capacities to build a better underwater speed and performance. As suggested by Veiga and Roig (2015), the underwater phases would require great skills in terms of technical control of the dolphin kick (Veiga and Roig, 2015), but these non-swimming parts are fully covered in apnea.

To our knowledge, no study had yet quantified the total non-swimming time during a swim race. This study results show that the elite swimmers have a greater percentage of non-swimming time during the race, approaching 40% of the total race time. Therefore, the technical skills of the non-swimming phases do not seem sufficient to optimize the performance and the individual strategy. Indeed, swimming 40% of the time underwater enhances the interest in the physiological repercussions on the swimmer, during the non-swimming phases in an apnea situation. It is well-known that apnea induces a typical cardiovascular response called a diving response, such as bradycardia (Foster and Sheel, 2005) and vascular adjustments (Joulia et al., 2009) that are important defense mechanisms of the body against hypoxia (Alboni et al., 2011). The diving response and skeletal muscle activity exert opposite influences on the heart and peripheral circulation (Wein et al., 2007; Alboni et al., 2011). The best swimmers are thus the ones with the longest apnea phase. They are likely managing more efficiently, the conflictual requirements between physical activity (i.e., bringing oxygen through the blood supply to the skeletal muscles) and apnea (i.e., bringing oxygen through to the heart and brain). Therefore, during the non-swimming apnea stages, since the conflict between the two inputs could appear, the physiological capacities involved might be more significant. Furthermore, apnea, by stopping the vital breathing function constitutes a psycho–physical stress coupled with repeated and prolonged apnea intervals and the intense dynamic exercises as suggested by Rodríguez-Zamora et al. (2014). This aspect needs to be considered when interpreting the differences noted regarding the non-swimming phases according to the performance levels.

The originality of this study is that it was performed during the competition conditions. On the other hand, the main limitation is the difficulty to measure the physiological data required in this ecological condition. Another limitation is the small differences observed between the UND50 and UP50, suggesting the necessity to introduce another lower-level swimming group such as regional level.

In conclusion, the current study presents new insights on the underwater parameters according to the level of swimming of the participants. Indeed, the international swimmers have covered more distance underwater, with a higher total non-swimming time, introducing new key data in the swimming performance. Coaches should, therefore, precisely monitor the race stages, with a focus on the underwater parts. Finally, various strategies of the underwater and surface parameters could be tested in order to reach optimal swimming performance. Thus, it is advisable to initiate further studies on the physiological dimension of apnea coupled with the biomechanical variables to investigate and understand the mechanisms involved during the non-swimming phases without neglecting the link with the swimming parts that precedes and follows the apnea but also to customize the training.

## Data Availability Statement

The original contributions presented in the study are included in the article/supplementary material, further inquiries can be directed to the corresponding author.

## Ethics Statement

Ethical review and approval was not required for the study on human participants in accordance with the local legislation and institutional requirements. Written informed consent for participation was not required for this study in accordance with the national legislation and the institutional requirements.

## Author Contributions

RP and AG conceived and designed the project. RP performed the data collection. RP, AG, and GP performed the data analysis and the interpretation of the data. RP, AG, GP, FJ, and CS contributed to the preparation of the manuscript. All authors contributed to the article and approved the submitted version.

## Conflict of Interest

The authors declare that the research was conducted in the absence of any commercial or financial relationships that could be construed as a potential conflict of interest.

## Publisher's Note

All claims expressed in this article are solely those of the authors and do not necessarily represent those of their affiliated organizations, or those of the publisher, the editors and the reviewers. Any product that may be evaluated in this article, or claim that may be made by its manufacturer, is not guaranteed or endorsed by the publisher.

## Abbreviations

UP50: French swimmers who performed the 100 m with a performance time upper to 50 s
UND50: French swimmers who performed the 100 m with a performance time under 50 s
SC: stroke count
SL: stroke length
SR: stroke rate
WORLD: world finalists.
